# Aerosol Delivery of Small Hairpin Osteopontin Blocks Pulmonary Metastasis of Breast Cancer in Mice

**DOI:** 10.1371/journal.pone.0015623

**Published:** 2010-12-22

**Authors:** Kyeong-Nam Yu, Arash Minai-Tehrani, Seung-Hee Chang, Soon-Kyung Hwang, Seong-Ho Hong, Ji-Eun Kim, Ji-Young Shin, Sung-Jin Park, Ji-Hye Kim, Jung-Taek Kwon, Hu-Lin Jiang, Bitna Kang, Duyeol Kim, Chan-Hee Chae, Kee-Ho Lee, Tae-Jong Yoon, George R. Beck, Myung-Haing Cho

**Affiliations:** 1 Laboratory of Toxicology, College of Veterinary Medicine, Seoul National University, Seoul, Republic of Korea; 2 Department of Nano Fusion Technology, Graduate School of Convergence Science and Technology, Seoul National University, Seoul, Republic of Korea; 3 Graduate Group of Tumor Biology, Seoul National University, Seoul, Republic of Korea; 4 Laboratory of Pathology, College of Veterinary Medicine, Seoul National University, Seoul, Republic of Korea; 5 Laboratory of Molecular Oncology, Division of Radiation Cancer Research, Korea Institute of Radiological and Medical Sciences, Seoul, Republic of Korea; 6 Department of Applied BioScience, CHA University, Seoul, Republic of Korea; 7 Division of Endocrinology, Metabolism, and Lipids, Emory University School of Medicine, Atlanta, Georgia, United States of America; Health Canada, Canada

## Abstract

**Background:**

Metastasis to the lung may be the final step in the breast cancer-related morbidity. Conventional therapies such as chemotherapy and surgery are somewhat successful, however, metastasis-related breast cancer morbidity remains high. Thus, a novel approach to prevent breast tumor metastasis is needed.

**Methodology/Principal Finding:**

Aerosol of lentivirus-based small hairpin osteopontin was delivered into mice with breast cancer twice a week for 1 or 2 months using a nose-only inhalation system. The effects of small hairpin osteopontin on breast cancer metastasis to the lung were evaluated using near infrared imaging as well as diverse molecular techniques. Aerosol-delivered small hairpin osteopontin significantly decreased the expression level of osteopontin and altered the expression of several important metastasis-related proteins in our murine breast cancer model.

**Conclusion/Significance:**

Aerosol-delivered small hairpin osteopontin blocked breast cancer metastasis. Our results showed that noninvasive targeting of pulmonary osteopontin or other specific genes responsible for cancer metastasis could be used as an effective therapeutic regimen for the treatment of metastatic epithelial tumors.

## Introduction

Osteopontin (OPN) is a secreted glycophosphoprotein that is believed to play a role in several apparently distinct cellular processes [1]. High-level OPN expression is one of the characteristics often associated with metastatic cancer cells [2]–[4]. As such, the metastatic activity of various cancer cells can be significantly inhibited by downregulation of OPN expression [5]–[8]. Many studies have shown a correlation between OPN and the progression and severity of many cancers, including breast, colon, lung, and prostate cancers. Particularly, elevated OPN levels and poor prognoses are highly associated with metastatic breast cancer [9], [10]. Moreover, the lungs are one of the most susceptible organs to breast cancer metastasis, which can differ in terms of evolution, treatment, morbidity, and mortality [11].

Recent studies have shown that RNA interference (RNAi)-mediated reduction of OPN expression may have therapeutic efficiency for many types of cancers [12.13]. RNAi, which can be induced in mammalian cells by small hairpin RNAs (shRNAs), is an evolutionarily conserved surveillance mechanism that targets double-stranded RNAs (dsRNAs) by sequence-specific silencing of homologous genes [14]. Gene therapy involving RNAi is an attractive strategy for the development of effective anticancer therapies due to its low level of toxicity. However, low gene transfection efficiency as well as technical difficulties associated with delivery regimen hinder the practical application of *in vivo* gene delivery. Especially, such problems clearly manisfest with regards to gene therapy to the lung [15].

To solve this, aerosol delivery distributes material uniformly and represents a noninvasive alternative for targeting genes to the lung. In fact, our group has demonstrated that viral as well as nonviral carrier-mediated gene delivery via inhalation may provide a means of treatment for a wide range of pulmonary disorders and offer numerous advantages over invasive modes of delivery [16], [17], [18].

Our successful noninvasive aerosol gene delivery system featuring shRNA-mediated RNAi has prompted us to develop a novel approach for the prevention of lung metastasis of breast cancer. In this study, shOPN was delivered into the lungs of mice having breast cancer via a nose-only aerosol delivery system in order to determine the preventive effects of suppressed OPN in breast cancer metastasis to the lung. Here, we report that aerosol-delivered shOPN blocked the metastasis of breast cancer to the lung. Our results envision that noninvasive targeting of pulmonary OPN or other specific genes responsible for cancer metastasis may constitute an effective therapeutic regimen for the treatment of metastatic epithelial tumors.

## Materials and Methods

### Lentivirus Construct for shOPN

The shRNA sequence targeting mouse OPN mRNA was designed. The sequence for knockdown of murine OPN expression was 5′-CGAGGTGATAGCTTGGCTTAT-3′. The scrambled sequence 5′-AAUCGCAUAGCGUAUGCCG-3′ was used as a control. shRNA was generated based on the above siRNA sequence and cloned into the pENTR/U6™ entry vector (Invitrogen, Carlsbad, CA, USA). Cassettes containing a U6 promoter and the shRNA target sequences were transferred into a lentivirus vector (pLenti6/BLOCK-iT™-DEST vector) by following the manufacturer's instructions. Recombinant lentiviral vectors were packaged using the ViraPower™ Lentiviral Packaging Mix (Invitrogen), after which the virus titer was determined using a HIV-1 p24 ELISA KIT (PerkinElmer Life Sciences, Boston, MA, USA).

### *In Vivo* Aerosol Delivery of Lentiviral shOPN

Five-week-old female Balb/c nude mice were purchased from Joongang Laboratory Animal Inc (Seoul, Korea). Animals were kept in the laboratory animal facility at a constant temperature and relative humidity of 23±2°C and 50±20%, respectively, under a 12 h light/dark cycle. Balb/c nude mice were injected with MDA-MB 231 cells (1×10^6^ cells/each in PBS) (Korean Cell Line Bank, Seoul, Korea) in the mammary fat pad. After 2 weeks [19], [20], the mice were divided into 3 groups (the control group was left untreated while the other two groups were exposed to aerosol containing lentivirus-shOPN or lentivirus-scrambled) [16], [17], [18]. The mice were then placed in a nose-only inhalation chamber and exposed to aerosol containing lentivirus solution (50 ml) containing 40 ng/ml of lentivirus-shOPN or scrambled control twice a week for 1 or 2 months, respectively. At the end of each time period, the mice were sacrificed and lungs were collected. During necropsy, neoplastic lesions on the lung surfaces were carefully counted under a microscope, as described by Singh et al [21]. All methods used in this study were approved by the Animal Care and Use Committee at Seoul National University (SNU-100303-1).

### Western Blot Analysis

Protein concentration of the homogenized lysates was measured using a Bradford kit (Bio-Rad, Hercules, CA, USA). Equal amounts (50 µm) of protein were separated by SDS–PAGE (10–15%) and transferred onto nitrocellulose membranes (Amersham Pharmacia, Cambridge, UK). After membranes were blocked in T-TBS containing 5% skim milk for 1 h, immunoblotting was performed by incubation overnight at 4°C with primary antibodies corresponding to OPN (R&D system, Minneapolis, MN, USA), CD44*v*6 (Millipore, Billerica, MA, USA), MMP-9 (Abcam, Billerica, MA, USA), MMP-2 (Novus, Littleton, CO, USA), VEGF (Santa Cruz, CA, USA), and PCNA (Santa Cruz, CA, USA) diluted 1∶1000 v/v in 5% skim milk, followed by incubation with second antibodies conjugated to horseradish peroxidase (HRP) for 3 h at room temperature or overnight at 4°C. After washing, the bands-of-interests were analyzed using a luminescent image analyzer LAS-3000 (Fujifilm, Tokyo, Japan).

### Histopathological Examinationand Immunohistochemistry (IHC)

After formalin-fixation, paraffin-embedded tissue sections were cut to 5 mm and transferred to Plus slides (Fisher Scientific, Pittsburgh, PA, USA). For histological analysis, tissue sections were stained with hematoxylin and eosin (H&E). For IHC, the tissues were deparaffinized in xylene (10 min ×2 times) and rehydrated through an alcohol gradient (each 5 min). The slides were then boiled in TE buffer (pH 9.0) for retrieval process, washed in tap water, and incubated in 3% hydrogen peroxide (AppliChem, Darmstadt, Germany) for 30 min to quench endogenous peroxidase activity. After washing in phosphate-buffered saline (PBS), the tissue sections were incubated with 3% bovine serum albumin in PBS for 1 h at room temperature in order to block unspecific binding sites. Primary antibodies were applied to tissue sections overnight at 4°C. On the second day, tissue slides were washed and incubated with secondary HRP-conjugated antibodies for 1 h at room temperature. After washing, tissue sections were counterstained with Mayer's Hematoxylin (DAKO, Carpinteria, CA, USA) and washed with xylene. Cover slips were then mounted using Permount (Fisher Scientific), and the slides were reviewed using a light microscope (Carl Zeiss, Thornwood, NY, USA). Staining of PCNA and OPN for quantification by IHC analysis was performed using the In Studio version 3.01 program (Pixera, San Jose, CA, USA). Staining intensity was assessed by counting the number of positive cells in randomly selected fields viewed at appropriate magnification through the objective lens.

### Wound Healing Assay

MDA-MB-231 cells were cultured to sub-confluence in 6-well plates. Streaks were made in the monolayer culture using 10 *µl* pipette tips. After washing away suspended cells, the remaining cells were treated with medium containing lentivirus-shOPN and lentivirus-scrambled. The progress of migration was photographed immediately 24 h after wounding.

### Statistical Analysis

The results of the Western blot analysis, IHC, and wound healing assay are expressed as the mean ± S.E.M of 3 independent experiments. Statistical analyses were performed following analysis of Student's *t*-test whenever the data consisted of only two groups. Quantification of Western blot analysis was performed using the Multi Gauge version 3.0 program (Fujifilm).

## Results

### Aerosol Delivery of Lentivirus-shOPN Decreases Breast Cancer Metastasis to Lung

**Figure 1:**
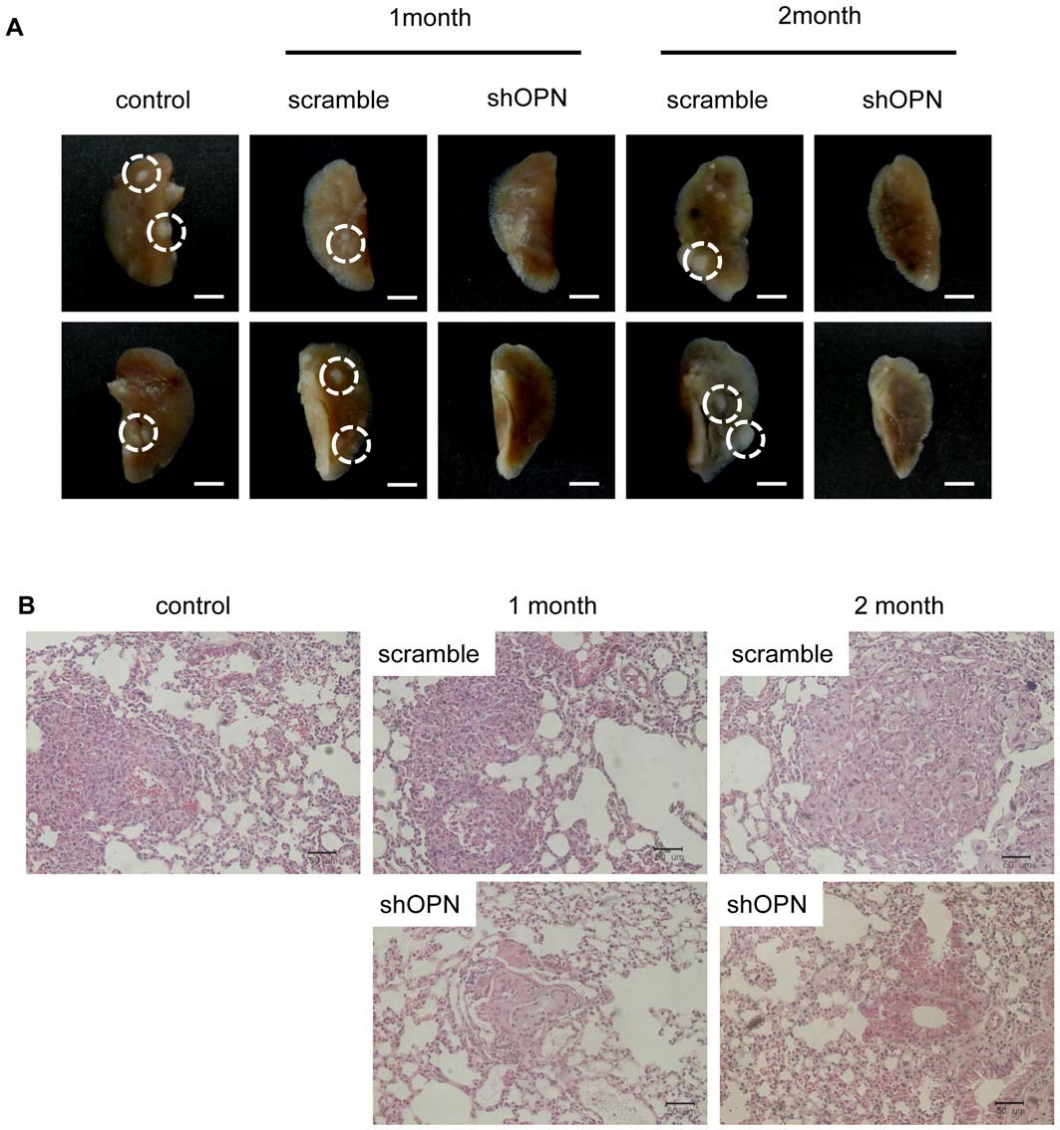
Lentivirus-shOPN decreases metastasis to the lung. (A) Gross morphological examination of the lung after aerosol delivery of shOPN for 1 or 2 months. White circles indicate neoplastic nodules observed in the lung. (B) Histopathological examination of lung after aerosol delivery of shOPN for 1 or 2 months. Magnification (×200) scale bar: 50 µm.

To determine whether or not aerosol delivery of shOPN affects breast cancer metastasis to the lung, lentivirus-shOPN was delivered twice a week for 1 or 2 months to breast cancer model mice. Gross morphological examination demonstrated that shOPN decreased lung tumor mass in the shOPN group compared to the control and scrambled group (Fig. 1A). Suppression of lung tumor metastasis was further confirmed by histolopathological analysis (Fig. 1B). Nodules formed in the lungs of the control and scrambled group were typical adenocarcinomas. However, the shOPN group demonstrated only a tumor mass, which was mostly likely an adenoma or hyperplasia. Such preventive effects against lung metastasis of breast cancer are clearly summarized in Table 1.

**Table 1:** Summary of tumor incidence in mouse model of metastatic breast cancer.

Group		Number of mouse	Tumor numbers/mouse			Adenocarcinomaincidence		Adenoma
			Total	>1.5 mm¶	<1.5 mm£	++$	+¥	
Control		4	6.25±0.87	1.5±1.73	4.75±1.94	2	1	1
1month	scrambled	4	6±2.61	2±1.83	4±1.08	1	1	2
	shOPN	4	2.13±1.03**	N.D.	2.13±1.03##	N.D.		2
2months	scrambled	4	9.25±0.87	2.5±1.29	6.75±0.87	1	1	2
	shOPN	4	2.38±0.63^##^ **	N.D.	2.38±0.63##	N.D.		2

Difinition of abbreviations: shOPN = small hairpin Osteopontin. N.D. = Not Detected.

Breast cancer model mice were exposed to lentivirus-shOPN twice a week for 1 or 2 months. After 1 or 2 months, the mice were sacrificed, lungs were collected, and the lesions on the lung surfaces were counted. Incidence and tumor number of lung mass were compared. Data were expressed as means ± S.E.M.

£ : Number of tumors of smaller than 1.5 mm in diameter.

¶ : Number of tumors of greater than 1.5 mm in diameter.

¥ : Adenocarcinoma grade was mild.

$ : Adenocarcinoma grade was moderate.

## : p<0.01 compared to scrambled group.

**: p<0.01 compared to control group.

### Aerosol Delivery of shOPN Decreases OPN Expression in the Lung

**Figure 2:**
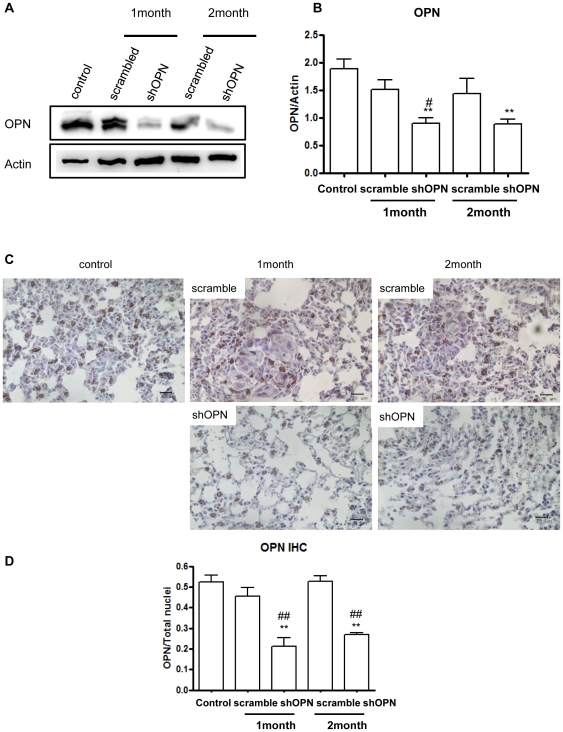
Effect of Aerosol-delivered shOPN on expression level of OPN. (A) Western blot analysis of OPN. (B) Densitometric analysis of OPN. (C) Immunohistochemical analysis of OPN. Magnification: ×400. Scale bar: 20 µm. (D) Comparison of OPN labeling index. Each bar represents the mean± S.E.M (n = 3). *Statistically significant different (P<0.05) compared to control group. **Statistically different (P<0.01) compared to control group. ^#^Statistically different (P<0.05) compared to scrambled control. ^##^Statistically different (P<0.01) compared to scrambled control.

To investigate the expression level of OPN in the lungs of breast cancer model mice after shOPN aerosol delivery, Western blot analysis was carried out. Lentiviral shOPN significantly decreased expression of OPN in the lungs of breast cancer model mice (Fig. 2A). Quantification of the bands-of-interest reconfirmed the Western blot results (Fig. 2B). IHC analysis also demonstrated that aerosol-delivered shOPN decreased expression of OPN compared to the control and scrambled group (Fig. 2C). Relative staining intensity for OPN clearly confirmed this finding (Fig. 2D).

### Aerosol Delivery of shOPN Suppresses Tumor Invasion and Angiogenesis

**Figure 3:**
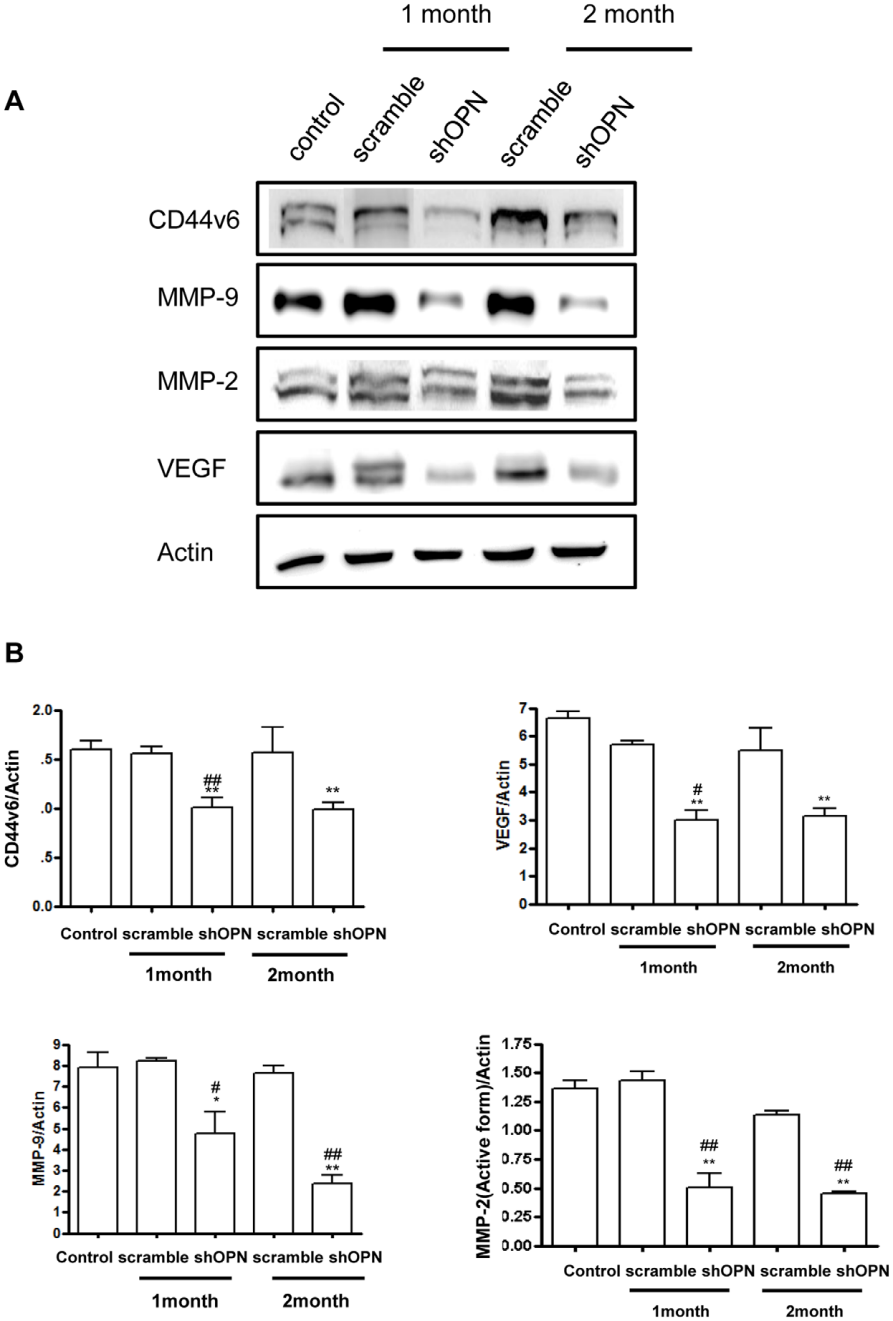
Western Blot Analysis of CD44v6, VEGF, MMP-9, and MMP-2. (A) Expression of CD44v6, VEGF, MMP-2, and MMP-9 in the lung. (B) Bands-of-interest were further analyzed by densitometry. Each bar represents the mean± S.E.M (n = 3). *Statistically different (P<0.05) compared to control group. **Statistically different (P<0.01) compared to control group. ^#^Statistically different (P<0.05) compared to scrambled control. ^##^Statistically different (P<0.01) compared to scrambled control.

Angiogenesis, the growth of new blood vessels, is known to promote tumor progression and metastasis [22]. Tumor invasion involves tumor cell penetration or infiltration into adjacent tissue and is related to the onset of metastasis [23]. In this study, we examined changes in the expression of angiogenesis- and invasion-related proteins by Western blotting. Our results clearly showed that aerosol delivery of shOPN significantly decreased the expression levels of CD44v6, VEGF, MMP-2, and MMP-9, as observed by densitometric analysis (Fig. 3A and 3B).

### Aerosol Delivery of shOPN Suppresses Cell Proliferation in the Lung

**Figure 4:**
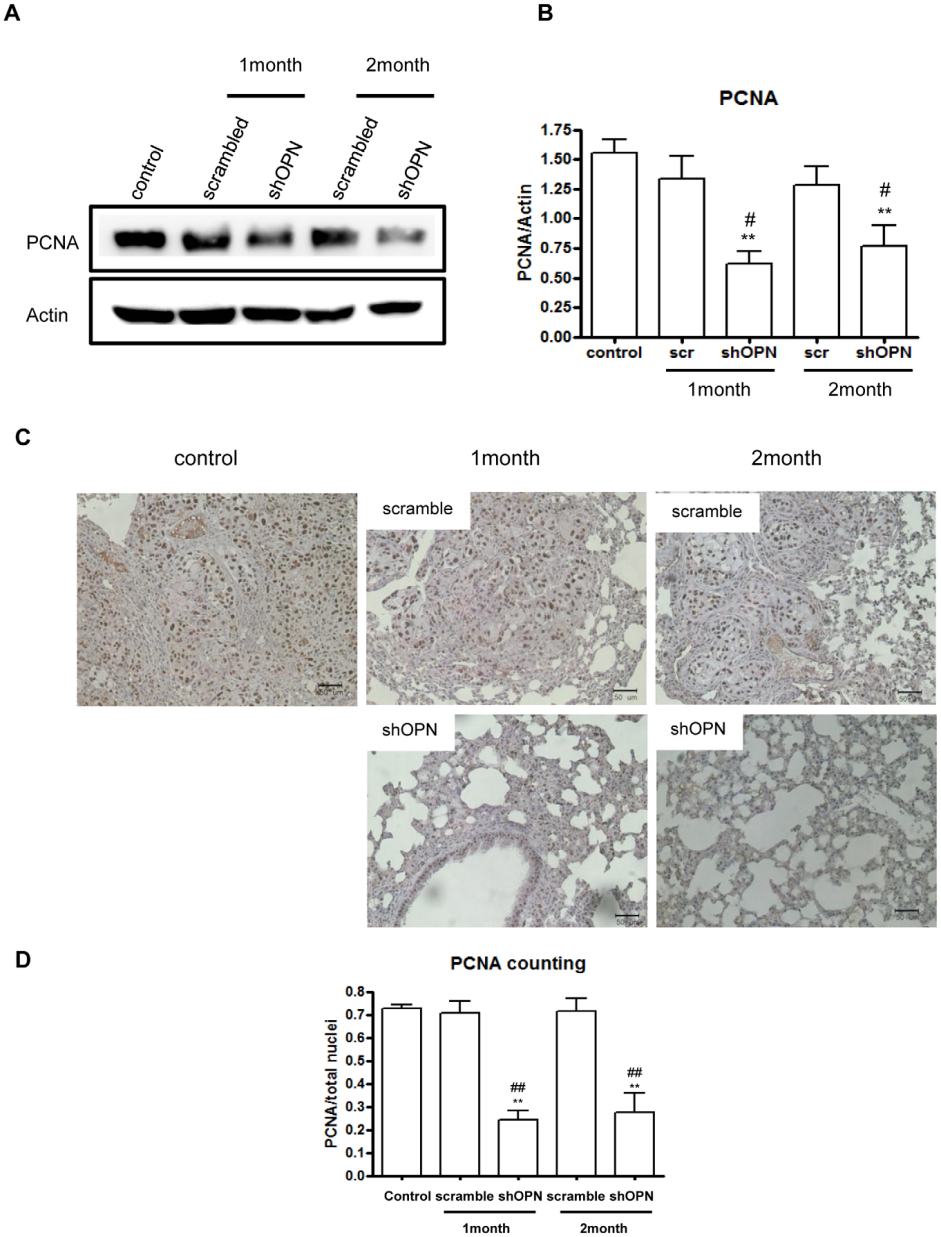
Effect of Aerosol-delivered shOPN on expression level of PCNA. (A) Western blot of PCNA. (B) Densitometric analysis of PCNA. (C) Immunohistochemical analysis of PCNA. Magnification: ×400. Scale bar: 20 µm. (D) Comparison of PCNA labeling index. Each bar represents the mean± S.E.M (n = 3). *Statistically different (P<0.05) compared to control group. **Statistically different (P<0.01) compared to control group. ^#^Statistically different (P<0.05) compared to scrambled control. ^##^Statistically different (P<0.01) compared to scrambled control.

Expression of proliferating cell nuclear antigen (PCNA) is increased in several types of cancers. Moreover, it has been postulated as a useful prognostic parameter for certain cancers [24]. Therefore, we determined the expression level of PCNA by Western blot analysis, which revealed that aerosol-delivered shOPN decreased PCNA expression in the lung of breast cancer model mice (Fig. 4A). Densitometric analysis further confirmed the Western blot results (Fig. 4B). Furthermore, IHC analysis showed that aerosol-delivered shOPN significantly decreased the expression level of PCNA compared to the control and scrambled group (Fig. 4C). The relative staining intensity also clearly confirmed this (Fig. 4D).

### shOPN Inhibits Cell Migration

**Figure 5:**
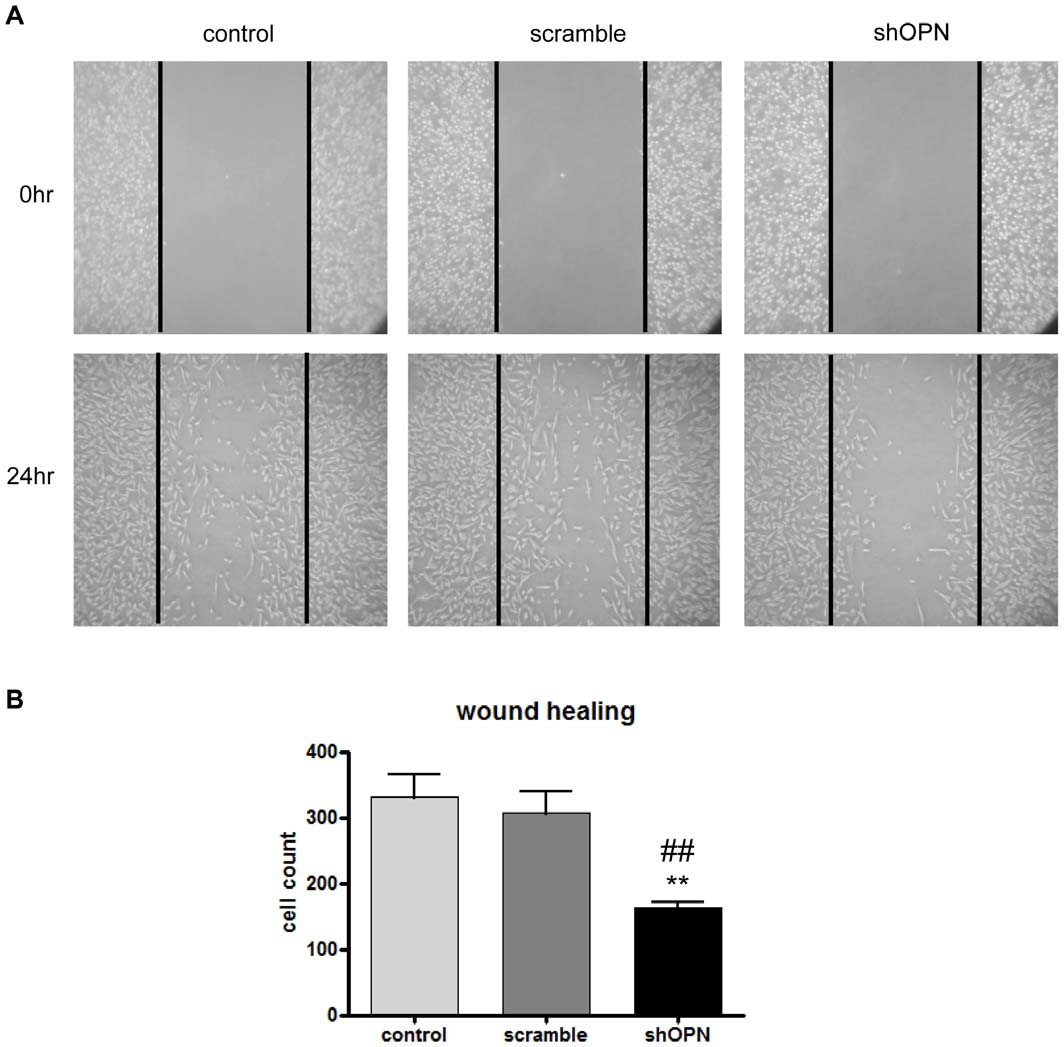
Wound Healing Assay. (A) Cell migration was inhibited by lentivirus-shOPN. MDA-MB 231 cells were grown up to 90% confluence, a single wound was made in the center of the cell monolayer and cell debris was removed by washing. After 24 h of incubation, the wound closure areas were visualized under an inverted microscope with a magnification ×100, (B) and the migrated cells were counted. **Statistically different (P<0.01) compared to control group. ^##^Statistically different (P<0.01) compared to scrambled control.

To investigate whether or not inhibition of OPN expression would reduce the migration of breast adenocarcinoma cells (MDA-MB231), wound healing assay was carried out. Our results showed that shOPN reduced the number of migrated cells after 24 h. Specifically, lentivirus-shOPN significantly inhibited motility of MDA-MB231 cells compared to the control and scrambled group (Fig. 5B).

## Discussion

Tumor metastasis may be divided into three different steps; separation of cancer cells from the primary tumor, circulation and migration of the cells through blood vessels to other organs, and finally, inhabitation of the cells in the target organs [25]. Many lines of evidence have reported that metastasis of breast cancer cells to the lungs probably occurs through the sentinel lymph node [26]. Therefore, metastasis to the lymph nodes can be a key prognostic factor of advanced disease status with the possibility of cancer cell migration from the original site to other more distant sites. [26], [27], [28]. In this study, with the aid of NIR imaging technology, we were able to detect the migration of cancer cells to the sentinel lymph node 2 weeks after injection of MDA-MB 231 cells into the mammary fat pad (Fig. S1). This finding is what caused us to perform aerosol gene delivery 2 weeks after cancer cell injection in order to focus on the 3^rd^ step of metastasis for preventing development of breast cancer cells to the lung.

Different approaches to gene delivery to the lung, such as intravenous injection and intratracheal instillation, have been reported in animal models [29.30]. These strategies are invasive or may not be appropriate for targeting pulmonary tissues. However, aerosol delivery is known to be an efficient method for delivering genes directly to the lungs [16], [17], [18]. Viral vectors have been demonstrated to be highly effective for gene delivery, and among these, lentiviral vectors provide for efficient gene transfer in cells and mediate stable, high-level transgene expression both *in vitro* and *in vivo* [17], [31], [32]. Therefore, in this study, we downregulated OPN expression in breast cancer model mice via aerosol delivery of lentiviral hOPN in order to investigate whether or not aerosol-delivered shOPN decreases metastatic spread of breast cancer cells to the lung.

OPN is a matricellular protein that is expressed in different tissues in the body, but mostly in bone. It is also produced by cancer cells and is known to play a crucial role in the growth, progression, and metastasis of cancer [33]. Actually, in breast cancer progression and metastasis, OPN is known as an important mediator, and it has been investigated as a potential therapeutic target in the treatment of breast cancer [34]. Patients of breast cancer suffering metastasis to the lung are faced with limited treatment options [35]. Since secreted phosphoproteins are readily accessible in the extracellular environment, OPN has gained much attention as an attractive therapeutic target for the blockade of tumor growth and metastasis. OPN binds to certain CD44 variants and integrin receptors [36], [37]. Expression of CD44 and its variant isoforms is associated with aggressive behavior of various tumors [38], [39]. Actually, CD44 variant 6 (CD44v6) is known to be overexpressed in lung and breast cancers [40], [41]. In this regard, our results showing significant suppression of CD44v6 by shOPN (Fig. 3) holds potential that shOPN delivery may be used in the treatment of breast cancer metastasis.

VEGF is a potent angiogenic cytokine with critical roles in tumor angiogenesis. VEGF produces may biological effects such as endothelial cell mitogenesis, migration, induction of proteinases leading to remodeling of the extracellular matrix, increased vascular permeability, and maintenance of the survival of newly formed blood vessels [42]. Overexpression of VEGF has been observed in a variety of human tumors; thus, increased VEGF expression in tumors has been established as an accurate biomarker of invasiveness, vascular density, metastasis, and recurrence [42], [43], [44]. Many researchers have demonstrated that co-expression of VEGF and OPN is well correlated with angiogenesis in patients with stage I lung adenocarcinoma [45]. More importantly, patients with VEGF-positive and OPN-positive lung adenocarcinoma suffer poor results due to increased postoperative metastasis and poor prognoses [46]. MMP-2 and MMP-9 are among the known factors that promote tumor cell motility, invasiveness, and epithelial-mesenchymal transition. MMPs are known to degrade dense extracellular matrix molecules (ECM) such as laminin, collagens, fibronectin, and proteoglycans in order to create space for tumor cell migration. Moreover, MMPs are known to activate certain growth factors associated with the ECM or cell surface [47]. Our results showed that aerosol delivery of shOPN significantly decreased the expression levels of CD44v6, VEGF, MMP-2, and MMP-9, strongly suggesting that suppression of OPN by lentiviral shOPN via aerosol can effectively inhibit migration, angiogenesis, and invasion of breast cancer cells into the lung. Epithelial cell proliferation in normal and cancerous lungs can be measured efficiently by quantifying the percentage of tumor cells positive for PCNA [48]. Our results showed that aerosol-delivered shOPN significantly decreased expression of PCNA. Therefore, combined with the effective suppression of several important angiogenic factors (Fig. 3), suppression of OPN by aerosol-delivered shOPN could probably prevent breast cancer cell metastasis into the lung.

In summary, our results demonstrated that aerosol-delivered shOPN suppressed the migration and invasion of breast cancer cells, leading to reduction of metastasis from the breast to the lung. Therefore, we suggest that aerosol gene delivery may prove to be a useful noninvasive model of gene delivery and that noninvasive targeting of pulmonary OPN or other genes responsible for cancer metastasis may be an effective therapeutic regimen for the treatment of metastatic epithelial tumors.

## Supporting Information

Figure S1 **Image of mouse model of metastatic breast cancer.** (A) Tagged MDA-MB 231 with NEO-LIVETM 797 (Biterials, Seoul, Korea) was injected into mouse mammary fat pad (red signals) and NIR signal detected using an image analyzer (MaestroTM, CRi, MA, USA). (B) In the mouse foot pad, NEO-LIVETM 675 (Biterials) was injected (green signals) and NIR signal detected using the same image analyzer. (C) Merged imaging picture. (D) Magnification image of indicated area (white square). (E) After NIR detection, sentinel lymph node was removed and stained with H&E. Red square indicates metastasis area of breast cancer cells (MDA-MB 231). Black dotted line indicates the boundary between invasion area and lymph node. C; Cortex, PF; Primary follicle. Scale bar: 50 μm (×200) (F) Magnified image of indicated area (red box of E). Scale bar: 20 μm (×400)
(TIF)
