# Supplementary figures and images for: Aerosol Delivery of Small Hairpin Osteopontin Blocks Pulmonary Metastasis of Breast Cancer in Mice
Source: PLoS One. 2010 Dec 22;5(12):e15623. doi: 10.1371/journal.pone.0015623 (PMC3008732; doi:10.1371/journal.pone.0015623)

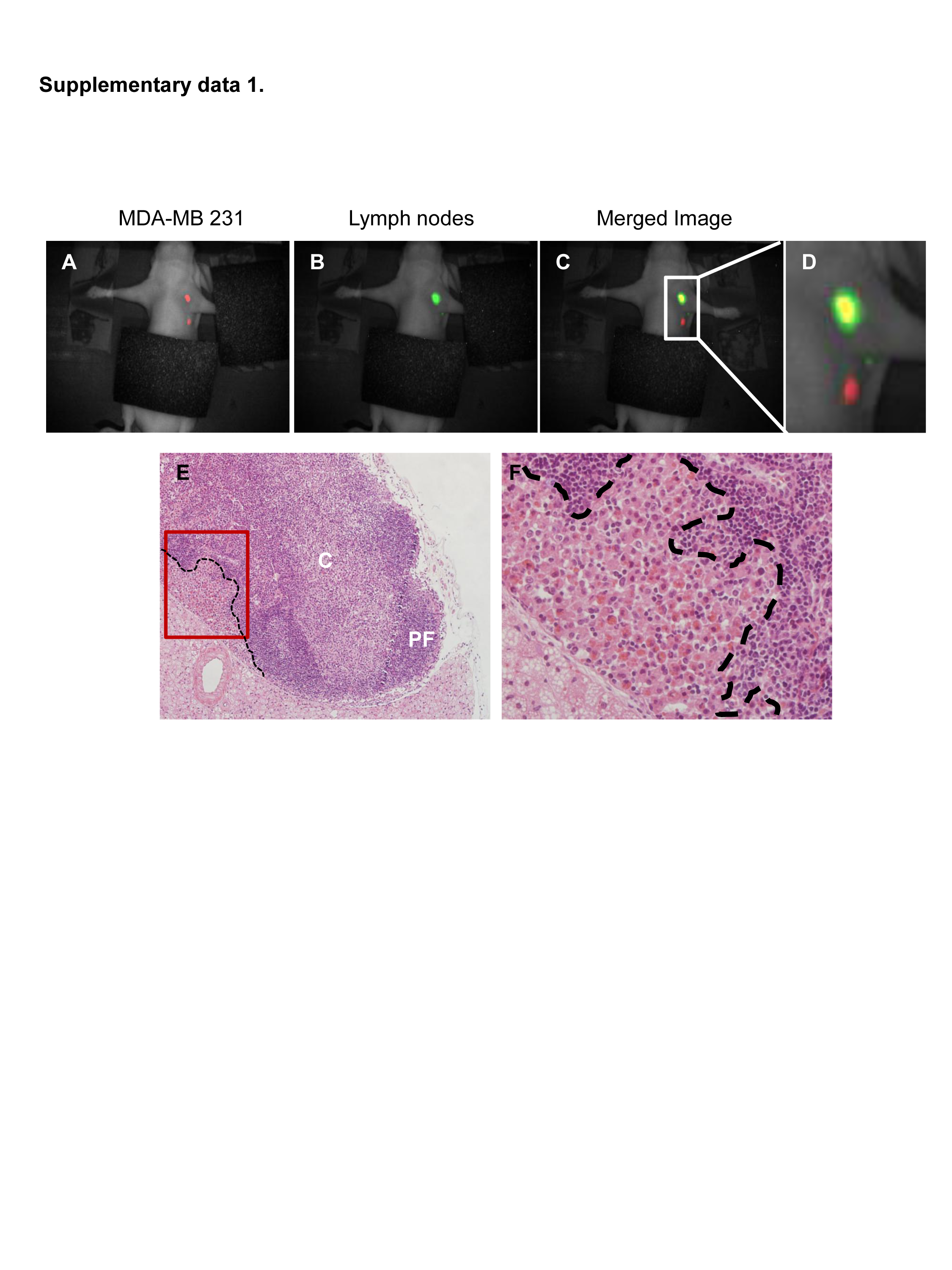

Supplement: Figure S1 — Image of mouse model of metastatic breast cancer. (A) Tagged MDA-MB 231 with NEO-LIVETM 797 (Biterials, Seoul, Korea) was injected into mouse mammary fat pad (red signals) and NIR signal detected using an image analyzer (MaestroTM, CRi, MA, USA). (B) In the mouse foot pad, NEO-LIVETM 675 (Biterials) was injected (green signals) and NIR signal detected using the same image analyzer. (C) Merged imaging picture. (D) Magnification image of indicated area (white square). (E) After NIR detection, sentinel lymph node was removed and stained with H&E. Red square indicates metastasis area of breast cancer cells (MDA-MB 231). Black dotted line indicates the boundary between invasion area and lymph node. C; Cortex, PF; Primary follicle. Scale bar: 50 μm (×200) (F) Magnified image of indicated area (red box of E). Scale bar: 20 μm (×400) (TIF) [file pone.0015623.s001.tif]
